# Supplementary material for: Empirical methods for controlling false positives and estimating confidence in ChIP-Seq peaks
Source: BMC Bioinformatics. 2008 Dec 5;9:523. doi: 10.1186/1471-2105-9-523 (PMC2628906; doi:10.1186/1471-2105-9-523)
Supplement: Additional File 1 — A variety of html documents from the USeq web site detailing the available applications, their best usage, output file type descriptions, command line menus, etc. [file 1471-2105-9-523-S1.zip › USeqUserGuides/JavaDoc.zip!/JavaDoc/util/bio/wrappers/Primer3Pick.html]

Primer3Pick


|  |  |  |
| --- | --- | --- |
| **Overview** \| **Package** \| **Class** \| **Use** \| **Tree** \| **Deprecated** \| **Index** \| **Help** | |  |
| **PREV CLASS**   **NEXT CLASS** | **FRAMES**    **NO FRAMES**     **All Classes** |  |
| SUMMARY: NESTED \| FIELD \| CONSTR \| METHOD | DETAIL: FIELD \| CONSTR \| METHOD |  |


---


## util.bio.wrappers Class Primer3Pick

```
java.lang.Object
  util.bio.wrappers.Primer3Pick
```

---

``` public class Primer3Pick extends java.lang.Object ```

Container for sequence used in picking primers with Primer3.

---

| **Field Summary** | |
| --- | --- |
| `static java.lang.String` | `header` |


**Constructor Summary**

`Primer3Pick(java.lang.String line)`


| **Method Summary** | |
| --- | --- |
| `static java.lang.String` | `getHeader()` |
| `java.lang.String` | `getLine()` |
| `java.lang.String` | `getResultSummaryLine()` |
| `java.lang.String` | `getSequence()` |
| `void` | `loadResults(java.util.HashMap results)`             Makes a tab delimited results line: PRIMER\_LEFT\_SEQUENCE PRIMER\_RIGHT\_SEQUENCE PRIMER\_LEFT\_TM PRIMER\_RIGHT\_TM PRIMER\_PRODUCT\_SIZE The user entered line If no results then leaves no picks message. |
| `static Primer3Pick[]` | `parseFile(java.io.File file)`             Parses a tab delimited file where the sequence to pick primers from is in column 0. |
| `void` | `setLine(java.lang.String line)` |
| `void` | `setResultSummaryLine(java.lang.String resultSummaryLine)` |
| `void` | `setSequence(java.lang.String sequence)` |

**Methods inherited from class java.lang.Object**

`equals, getClass, hashCode, notify, notifyAll, toString, wait, wait, wait`

**Field Detail**

### header

```
public static final java.lang.String header
```

**See Also:**: Constant Field Values


**Constructor Detail**

### Primer3Pick

```
public Primer3Pick(java.lang.String line)
```


**Method Detail**

### loadResults

```
public void loadResults(java.util.HashMap results)
```

:   Makes a tab delimited results line:
    PRIMER\_LEFT\_SEQUENCE
    PRIMER\_RIGHT\_SEQUENCE
    PRIMER\_LEFT\_TM
    PRIMER\_RIGHT\_TM
    PRIMER\_PRODUCT\_SIZE
    The user entered line
    If no results then leaves no picks message.

---


### parseFile

```
public static Primer3Pick[] parseFile(java.io.File file)
```

:   Parses a tab delimited file where the sequence to pick primers from is in column 0.

---


### getLine

```
public java.lang.String getLine()
```

---


### getSequence

```
public java.lang.String getSequence()
```

---


### getHeader

```
public static java.lang.String getHeader()
```

---


### getResultSummaryLine

```
public java.lang.String getResultSummaryLine()
```

---


### setResultSummaryLine

```
public void setResultSummaryLine(java.lang.String resultSummaryLine)
```

---


### setLine

```
public void setLine(java.lang.String line)
```

---


### setSequence

```
public void setSequence(java.lang.String sequence)
```


---


|  |  |  |
| --- | --- | --- |
| **Overview** \| **Package** \| **Class** \| **Use** \| **Tree** \| **Deprecated** \| **Index** \| **Help** | |  |
| **PREV CLASS**   **NEXT CLASS** | **FRAMES**    **NO FRAMES**     **All Classes** |  |
| SUMMARY: NESTED \| FIELD \| CONSTR \| METHOD | DETAIL: FIELD \| CONSTR \| METHOD |  |


---
